# Silver Nanoparticle Films Obtained by Convective Self-Assembly for Surface-Enhanced Raman Spectroscopy Analyses of the Pesticides Thiabendazole and Endosulfan

**DOI:** 10.3389/fchem.2022.915337

**Published:** 2022-06-29

**Authors:** I. A. Brezestean, N. Tosa, A. Falamas, D. Cuibus, C. M. Muntean, A. Bende, B. Cozar, C. Berghian-Grosan, C. Farcău

**Affiliations:** ^1^ National Institute for Research and Development of Isotopic and Molecular Technologies, Cluj-Napoca, Romania; ^2^ Biomolecular Physics Department, Babes-Bolyai University, Cluj-Napoca, Romania

**Keywords:** nanoparticle, colloid, convective self-assembly, SERS (surface enhanced Raman scattering), endosulfan, thiabendazole

## Abstract

Pesticides pose a great threat to human health and their rapid detection has become an urgent public safety issue engaging the scientific community to search for fast and reliable detection techniques. In this context, Surface Enhanced Raman Spectroscopy (SERS) has emerged as a valuable detection and analysis tool due to its high sensitivity and selectivity, proving its suitability for the food industry and environmental monitoring applications. Here, we report on the fabrication of colloidal silver nanoparticle (AgNP) films by convective self-assembly (CSA) on solid planar substrate and their use for the SERS analyses of two types of pesticides, the fungicide thiabendazole (TBZ) and the insecticide α-endosulfan (α-ES). Electron microscopy shows that these nanoparticle films are dense, highly compact, and uniform across several mm^2^ areas. The SERS efficiency of the fabricated AgNP films is evaluated using a well-known Raman probe, p-aminothiophenol, for multiple excitation laser lines (532 nm, 633 nm, and 785 nm). The films exhibit the largest SERS enhancement factors for 785 nm excitation, reaching values larger than 10^5^. Thiabendazole could be readily adsorbed on the AgNPs without any sample surface functionalization and detected down to 10^−6^ M, reaching the sub-ppm range. Endosulfan, a challenging analyte with poor affinity to metal surfaces, was captured near the metal surface by using self-assembled alkane thiol monolayers (hexanethiol and octanethiol), as demonstrated by the thorough vibrational band analysis, and supported by density functional theory (DFT) calculations. In addition, principal component analysis (PCA) based on SERS spectra offers significant leverage in discrimination of the molecules anchored onto the metallic nanostructured surface. This present study demonstrates the utility of self-assembled colloidal nanoparticle films as SERS substrates for a broad range of analytes (para-aminothiophenol, thiabendazole, α-endosulfan, and alkanethiols) and contributes to the development of SERS-based sensors for pesticides detection, identification and monitoring.

## Introduction

Pesticides are among the most dangerous chemicals that damage the environment and require strict monitoring (Europäische Kommission, 2007). Even though they play a crucial role in protecting cultures and improving yields, incorrect application of pesticides can lead to large amounts of residues in the environment, including surface waters, becoming thus a threat for the population (Popp et al., 2013). The protection of water resources has thus been identified as priority by the European Commission. Raman spectroscopy represents a relatively simple, rapid and portable method for detecting the structure and composition of environmental samples. Considering its limitation in terms of weak Raman signal, the method is however not suitable for detecting trace amounts in samples. Surface Enhanced Raman Spectroscopy (SERS) (Bernat et al., 2019) on the other hand, enhances the Raman scattering of molecules adsorbed at the surface of metal nanostructures and can be used to detect low concentrations of pesticides in the environment (Halvorson and Vikesland, 2010) and food industry (Müller et al., 2014; Li et al., 2018a). In principle, the SERS effect is obtained when an analyte is adsorbed on or in the nanometric proximity to a prepared metal surface (i.e., SERS substrate) (Bantz et al., 2011), which is usually composed of nanostructured noble metals (e.g., Ag, Au, Cu). The localized surface plasmon resonance (LSPR) effect, resulting from the collective oscillations of the conduction electrons localized at the surface of the nanostructures (Granger et al., 2013; Shiohara et al., 2014), leads to the generation of strong electromagnetic fields and induces an amplified Raman scattering by the analyte. In other words, the Raman excitation laser generates plasmonic excitations on the metal surface, which interact with the analyte on the surface, commonly providing a 10^6^–10^7^ increase in scattering efficiency over normal Raman scattering. The SERS technique has been employed in multiple research studies and has yielded promising results as a method suitable for detecting and monitoring pesticides even at trace levels (Kubackova et al., 2015).

Various materials and morphologies have been explored for pesticide detection, including colloidal gold (Au) and (Ag) nanoparticles (Luo et al., 2016; Dube and Rawtani, 2021), Ag nanoflowers (Zhang et al., 2018), Au/Ag nanorods (Luo et al., 2016; Li et al., 2018b), Ag–Au core-satellite nanostructures (Guo et al., 2020), Ag decorated sandpaper (Fan et al., 2014), substrates manufactured using different techniques, e.g., e-beam lithography (Abu Hatab et al., 2008) (EBL), ultraviolet lithographic techniques (Zhang et al., 2017), and low concentrations of pesticides up to 10^−10^ M could be detected (Sitjar et al., 2019). Solid SERS substrates fabricated by advanced nanolithography methods such as EBL are excellent in terms of control over the nanostructure morphology and tunability of the optical response, but fail in producing nanoscale gaps, necessary for extreme SERS enhancements.

Au and Ag colloidal nanoparticles, generally having diameters in the range 10–200 nm, are easy to synthesize and can be used for the detection of pesticide residues and biotoxins (Chamuah et al., 2018; Zhou et al., 2021). However, they have some disadvantages like limited reproducibility, stability, or uncontrolled aggregation induced by analytes. An approach to overcome some of these disadvantages and benefit from the advantages of colloidal nanoparticles, is based on the development of SERS-active substrates made of colloidal metal nanoparticles deposited as films on solid supporting substrates (Farcau et al., 2013; Zhang et al., 2019; Wang et al., 2021). When these films exhibit a compact packing and good surface uniformity, they represent a very good alternative to nanostructured films obtained by advanced nanolithography, offering a good-enough reproducibility and very often even higher SERS enhancements.

Thiabendazole (TBZ), a benzimidazole derivative, is one of the widely used pesticides, also used as a fungicide to prevent mold, rot and fruit scorch. Although TBZ has low toxicity compared to other pesticides, it is associated with adverse effects including nephrotoxicity, hepatotoxicity, carcinogenicity, and teratogenicity (Fu et al., 2019). To measure TBZ residues in food samples, a SERS method combined with a homogeneous and reusable gold nanorod array (GNR) substrate has been proposed (Fu et al., 2019). SERS has also been applied as an analytical technique to detect thiabendazole (TBZ) at low concentrations using Ag colloid (Müller et al., 2014; Oliveira et al., 2020). Furthermore, a plasmonic array of silver-coated gold nanoparticles (Au@Ag NP) packed with a ternary film was fabricated and applied as a stable and high-performance SERS chip for highly sensitive detection of thiabendazole (TBZ) residues in fruit juices (Wang et al., 2019). SERS was applied to study the adsorption of thiabendazole (TBZ) on a silver mirror (Kim et al., 2009). Endosulfan-(ES) is a broad-spectrum organochlorine pesticide that has been used on cereals, fruits, vegetables and cotton since the 1950s (Maier-Bode, 1968; Schmidt et al., 2014) and is also one of the most stable substances detected in the environment worldwide. It belongs to the category of persistent organic pollutants (POPs) (United Nations Targets Widely-Used Pesticide Endosulfan for Phase Out; United Nations Environment Programme: Geneva, 2011 (United Nations, 2011) because of its bioaccumulation, its resistance to long-range transport and its negative effects on human health, ecosystems and aquatic systems (Weber et al., 2010; Becker et al., 2011). In addition, the organochlorine pesticide ES was detected for the first time using surface-enhanced Raman scattering at low concentrations (Guerrini et al., 2008), the bis-acridinium dicationlucigenine was successfully used as a molecular assembler in the functionalization of metal nanoparticles to facilitate the approach of the pesticide to the metal surface (Guerrini et al., 2008). The detection of organochlorine pesticides is still very challenging, due to the poor affinity to metal surfaces. Some of the few approaches to tackle this problem are presented in a recent review (Moldovan et al., 2021). Despite the promising existing results, there is still a need to develop low-cost, easy and reliable approaches for pesticide detection. In order to obtain reproducible results, solid substrates, e.g., films that are readily available, functionalized, cheap and efficient should be used for SERS detection.

In this work, we report on the fabrication of colloidal silver nanoparticle (AgNP) films by the convective self-assembly (CSA) of silver nanoparticles synthesized by the Lee and Meisel method (Lee and Meisel, 1982). We thus propose a kind of SERS substrate which is fabricated by depositing colloidal nanoparticles directly from suspensions onto solid substrates. After a morphological characterization by electron microscopy, the films are evaluated as a SERS substrate with the well-known para-aminothiophenol (p-ATP) molecule, by using three excitation laser lines. Subsequently, the possibility of using the fabricated AgNP films as substrates for SERS detection and characterization of two types of pesticides, thiabendazole and α-endosulfan is explored. The appropriateness of the AgNP films for specific surface functionalization protocols necessary for better anchoring of pesticides near the metal surface was also demonstrated. Specifically, two types of thiol molecules, hexanethiol (HT) and octanethiol (OT), were used in an attempt to improve ES-to-metal surface interaction. Additionally, it is observed that the SERS signals of HT and OT on the AgNP films offer clear spectral differences, useful for their differentiation, despite the similarity of these two molecules. Theoretical tools such as Density Functional Theory (DFT) for Raman band assignment and Principal Component Analysis (PCA) for analyzing acquired SERS data sets are also used in this study towards pesticide identification.

## Experimental/Methods

### Materials

The certified analytical grade reagents such as silver (I) nitrate 99.0% (AgNO_3_), trisodium citrate dihydrate (C_6_H_5_Na_3_O_7_·2H_2_O), denoted citrate, para-Aminothiophenol 97% (H_2_NC_6_H_4_SH), denoted p-ATP, 1-Hexanethiol 95% (C_6_H_13_SH), denoted HT, 1-Octanethiol 98.5% (C_8_H_17_SH), denoted OT, α-Endosulfan (6,7,8, 9,10,10-hexachloro-1,5,5a,6,9,9a-hexahydro-6,9-methano-2,4,3-benzodioxathiepine-3-oxide), PESTANAL 98% (C_9_H_6_Cl_6_O_3_S), denoted ES, and Thiabendazole [2-(4-thiazolyl)benzimidazole], PESTANAL 99% (C_10_H_7_N_3_S), denoted TBZ, were commercially obtained from Merck (Sigma-Aldrich) and used as received. Ethanol absolute (Merck) was used to prepare the molar stock solutions of 10^−2^ M (4-ATP and ES) and 5·10^−3^ M (HT, OT, and TBZ), the molar solutions of the related concentrations of 10^−4^ M (p-ATP and ES) and 5·10^−4^ M (HT and OT), 5·10^−4^–10^−6^ M (TBZ), as well as to wash the samples upon deposition. Milli-Q water, obtained in laboratory, was used to prepare all aqueous solutions and to wash the Ag NPs before film deposition, respectively.

### AgNPs Synthesis and Characterization

AgNPs were prepared according to the classical Lee and Meisel synthesis approach (Lee and Meisel, 1982). Briefly, 100 ml of aqueous solution containing 17 mg of AgNO_3_ salt was heated to the boiling point (100°C) followed by the dropwise addition of 2 ml of 1% trisodium citrate solution during constant stirring. The mixture was thermally treated for 45 min at 100°C and allowed to cool down in ambient conditions. The freshly prepared colloidal AgNPs solution shows milky-grey colour. Their optical extinction spectrum was measured by a Jasco V550 UV-VIS spectrophotometer. For the convective self-assembly process, the concentration of the colloid was increased about 20 times by centrifugation and resuspension.

### Fabrication of Nanoparticle Films by Convective Self-Assembly

Microscope glass slides were cleaned with acetone, methyl alcohol and isopropyl alcohol. After washing, they were dried with nitrogen and subjected to UV-ozone treatment for 20 min to obtain a hydrophilic surface. Colloidal nanoparticle films were then prepared using the convective self-assembly (CSA) method with a home-made equipment. An amount of nanoparticle suspension (∼10 μl) was injected between the substrate and a deposition blade that is positioned near the substrate. By substrate translation, due to the evaporation of water causing particles flow from the solution to the meniscus, nanoparticle films were obtained. All samples were prepared in ambient conditions.

### Sample Preparation for SERS Measurements

For SERS measurements, samples were immersed for 24 h in 10^−4^ M ethanol solution of p-aminothiophenol (p-ATP). Subsequently, samples were washed several times with alcohol to remove any unabsorbed molecules. The SERS signal of films fabricated by self-assembly with Ag NPs immersed in p-ATP was tested for three different excitation laser lines. AgNP films used for the detection of thiabendazole (TBZ) pesticide were prepared by immersing them overnight in 5*10^−4^ M TBZ solution, followed by rinsing. The ES-coated AgNP films functionalized with HT and OT, respectively, were prepared in a two-stages process. First, the AgNP films were completely immersed into 5·10^−4^ M ethanol solutions of HT and OT, respectively, prepared by dilution from their stock solutions of 5·10^−3^ M concentration. After 24 h, the films were removed from their corresponding thiol solutions and washed with absolute ethanol to remove the unbound molecules from the functionalized films (HT—AgNP film and OT—AgNP film) were allowed to dry in air, then stored into individual sealed boxes in dark, to be used as samples, but also as references for the following measurements. In the second step, the thiol-functionalized AgNP films were immediately immersed into 10^−4^ M ethanol solutions of ES, prepared by dilution from its stock solutions of 10^−2^ M concentration. After 24 h the films were removed from the ES solutions and allowed to dry in the air, then stored into individual sealed boxes in dark until measurements were performed (ES—HT—AgNP film and ES—OT—AgNP film). During both deposition stages, the recipients were kept at room temperature in dark.

### Raman/SERS Measurements and Data Processing

Raman and SERS spectra at 785 nm excitation wavelength were recorded with a JASCO NRS-3300 Raman Spectrometer equipped with a CCD detector (−69°C) using an Olympus UMPLFL ×100 objective, 600 lines/mm grating, 0.2 × 6 mm slit at 2.8 mW power and optical density (OD)2 attenuation. The spectra were recorded in 78–1823 cm^−1^ frequency range with a resolution of 0.1 cm^−1^ at 10 s exposure time and 10 accumulations per sample. The calibration was performed using a sharp peak of Si at 521 cm^−1^. Raman and SERS spectra were recorded also using a Renishaw InVia Reflex Raman confocal spectrometer equipped with the 633 nm and a Leica microscope equipped with a ×100 objective. The signal was collected in the range 100–1600 cm^−1^ using a filter with edge >100 cm^−1^. The spectral resolution was 1 cm^−1^. The acquisition conditions were: 10 s integration time, one accumulation, 1% laser power of 17 mW total power. We also used a portable spectrometer, i-Raman Plus from B&W TEK, equipped with a 532 nm laser line (total power 50 mW) connected *via* an optical fiber to a BW-TEK optical microscope equipped with a ×20 objective. The acquisition conditions were 10 s integration time, five accumulations, and 10% laser power.

### Electron Microscopy

Scanning Electron Microscopy (SEM) images and Energy Dispersive X-ray (EDX) spectrum were obtained using a cold field emission Hitachi SU8230 system operating at accelerating voltage up to 30 kV and magnifications up to ×150000. The samples were coated before analysing with a 5-nm conductive gold layer to increase the contrast level in the secondary electron images. Scanning transmission electron microscope (STEM) images were recorded using a Hitachi HD-2700, equipped with a cold field emission gun, working at 200 kV acceleration voltage and designed for high-resolution (HRTEM) imaging with a resolution of 0.144 nm. A droplet of silver colloidal suspension (each sample) was deposited and dried on a copper grid coated by a thin carbon film prior to the STEM analysis. The secondary electrons signal (surface topography) as well as the transmitted electrons signal (morphology and composition) were employed to carry out the morphological investigations.

### DFT Calculations

The equilibrium geometries, normal mode vibrational frequencies and the corresponding Raman intensities were obtained in the framework of the density functional theory (DFT) considering the ωB97X exchange-correlation functional (Chai and Head-Gordon, 2008) combined with the D3-type empirical dispersion correction scheme (Grimme et al., 2010; Lin et al., 2013) and applying the minimally augmented (Zheng et al., 2011) ma-def2-TZVPP triple-zeta basis set of the Karlsruhe group (Weigend and Ahlrichs, 2005) as implemented in the ORCA program suite (Neese, 2012, Neese, 2018). The RIJCOSX approximation (Neese et al., 2009) designed to accelerate Hartree-Fock and hybrid DFT calculations were considered together with the Def2/J (Weigend, 2006) auxiliary basis set for Coulomb fitting.

## Results

**FIGURE 1:**
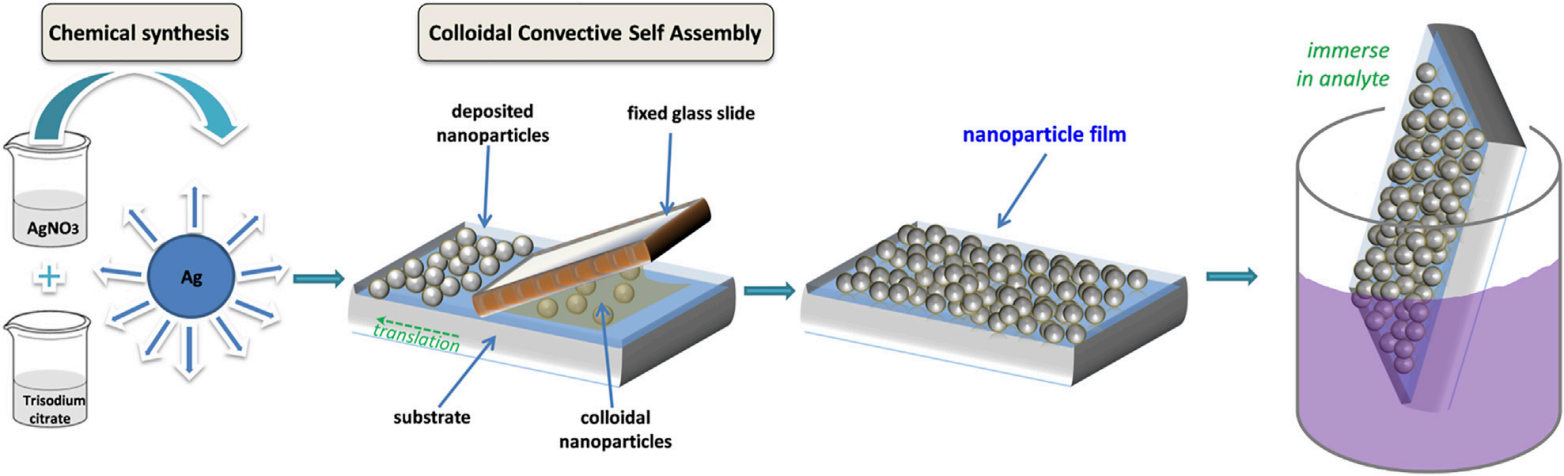
Schematized protocol for fabrication of AgNP films for use as SERS substrate: AgNP are chemically synthesized, then assembled into films on solid substrates; the films are then immersed in analyte solutions before SERS measurements.

The protocol proposed here for development of colloidal nanoparticle-based SERS substrates is schematically presented in Figure 1. First, the chemical synthesis of colloidal silver nanoparticles takes place; we used here the now classical Lee and Meisel method, but any synthesis in aqueous medium could be further used. Next, the colloidal AgNPs are assembled into films by the CSA method. The assembly process relies on the evaporation of water near the triple-contact line (meniscus), which induces a flux of particles towards this region. By controlling the shape of the water droplet by means of the fixed rectangular glass slide, the particles are deposited along a straight line on the substrate. By adjusting the translation speed of the substrate, the film growth rate can be regulated. After allowing the nanoparticle films to dry in air, these are then immersed in solutions containing the analytes under study, and then used for SERS measurements.

### AgNP Colloid Characterization

**FIGURE 2:**
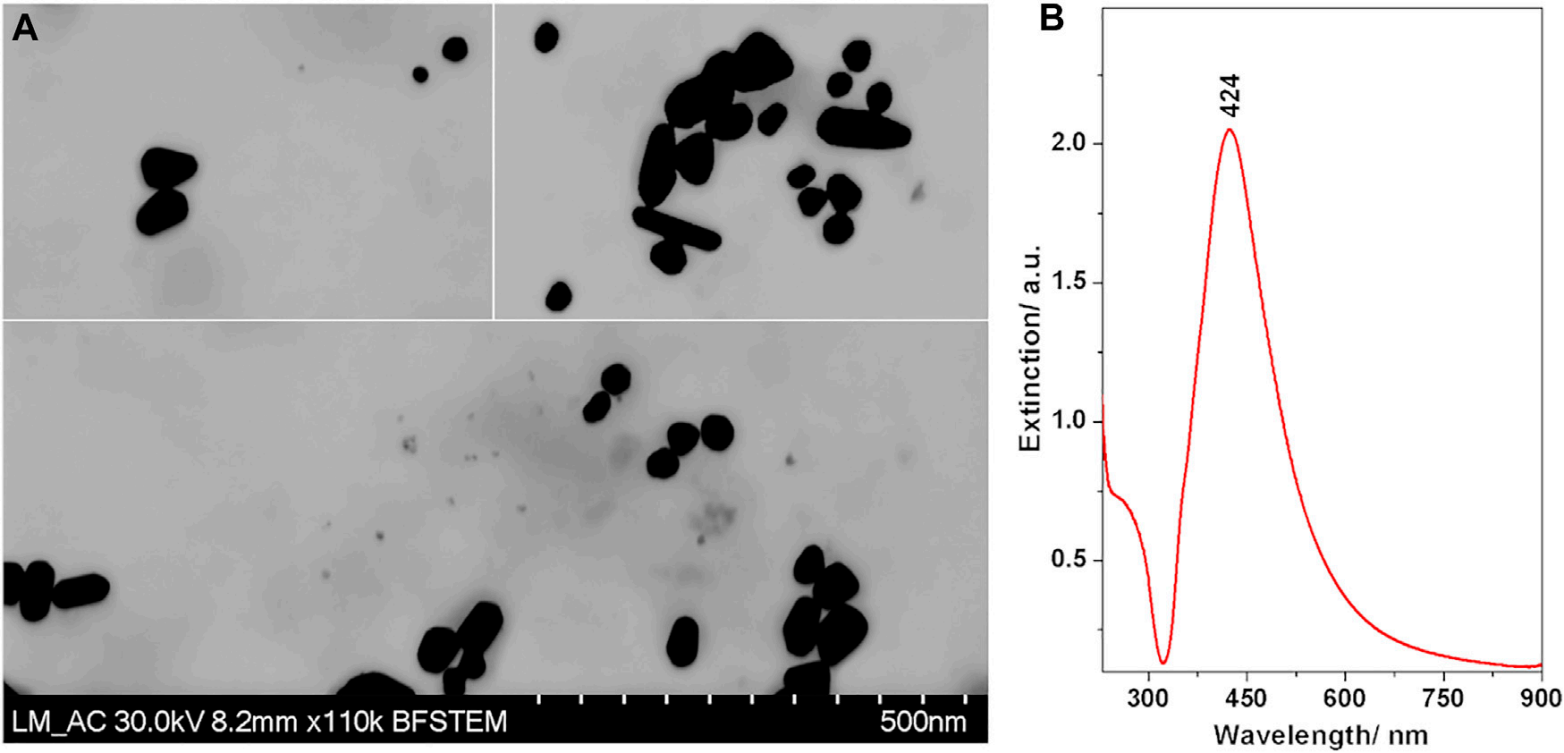
**(A)** TEM images of the Ag nanoparticles. **(B)** Extinction spectrum of the synthesized Ag nanoparticle colloidal suspension.

TEM images of the prepared colloidal AgNPs are presented in Figure 2A. Besides the spherical shaped particles, many possessed an asymmetric rod-like shape. The size histogram indicated a bimodal size distribution, the majority of particles having sizes around 40 nm, with an additional population sized around 60 nm (Supplementary Material).

The UV/VIS extinction spectrum (Figure 2B) exhibited the characteristic band attributed to localized surface plasmon resonance with a maximum at 424 nm. The band was broad with a strong asymmetry towards the infrared, which is correlated well with the non-spherical shapes of the nanoparticles observed in the TEM images.

### AgNP Film Characterization

**FIGURE 3:**
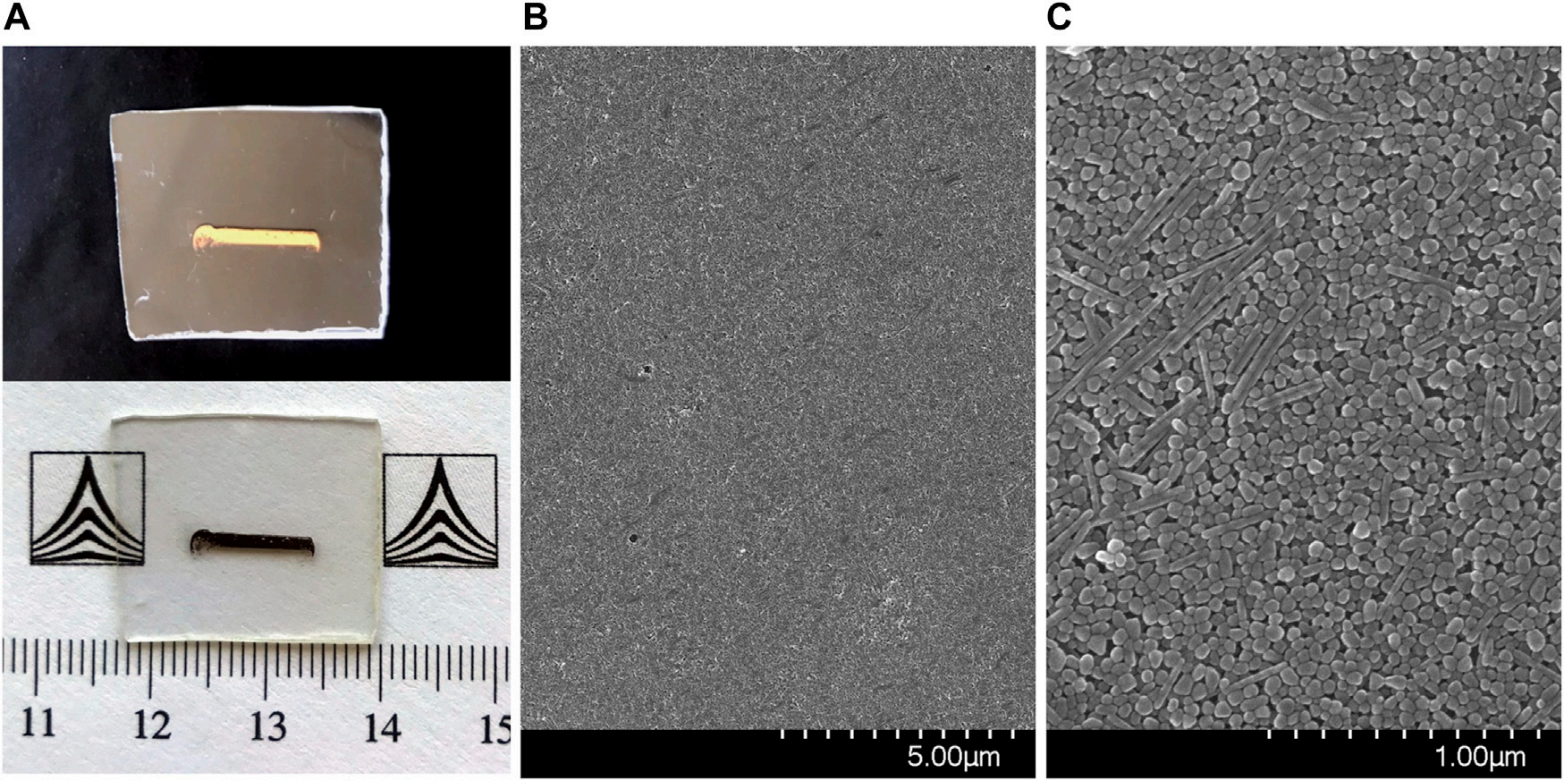
**(A)** Photographs of the AgNP film under different background and lighting conditions; **(B,C)** SEM images of the AgNP films.

One of the advantages of the CSA approach for the deposition of nanoparticle films is the fact that it allows to directly form nanoparticle films onto a wide range of solid, planar substrates. The areas which can be coated can scale up to cm^2^, depending on the particular setups in use. In our case, the fabricated films were rectangular shaped, covering areas of about 2 × 10 mm^2^. Photographs of a typical sample are displayed in Figure 3A under different lighting and background conditions. The shiny, gold-like appearance under specular light indicated a highly compact arrangement of the nanoparticles within the films.

**FIGURE 4:**
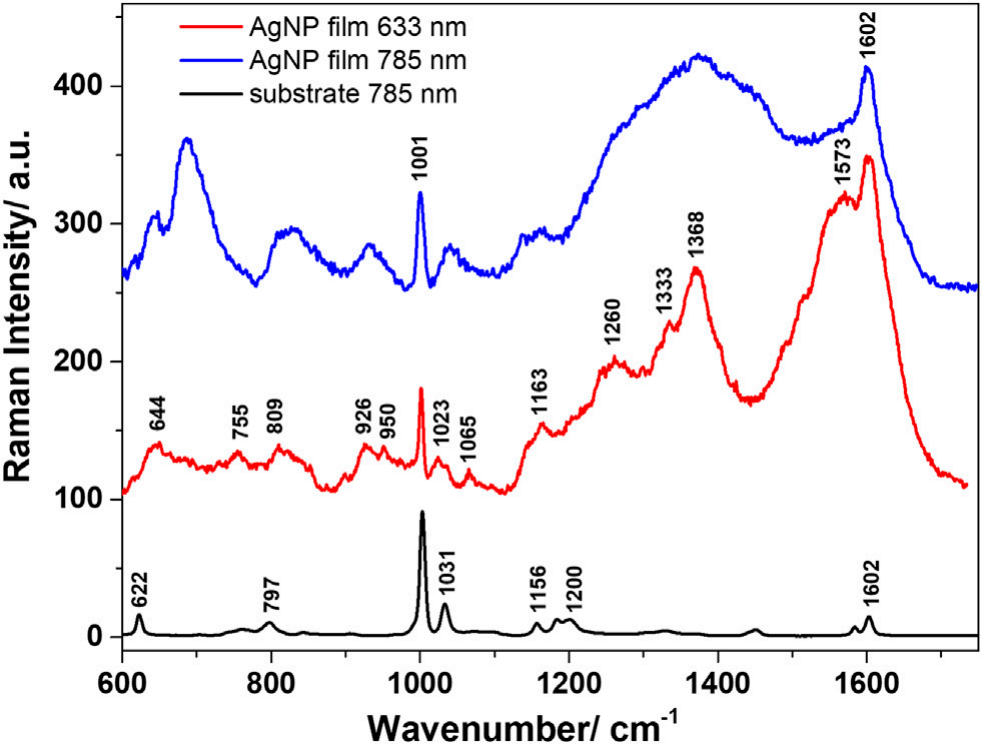
Average Raman spectra characteristic to the polystyrene substrate and the AgNP film obtained with 633 nm, and 785 nm excitation.

The morphology of the self-assembled films of Ag nanoparticles was further characterized by SEM, images being shown in Figures 3B,C. A highly compact packing of the nanoparticles can be observed, along with a very good uniformity across the sample surface. Before proceeding to SERS analyses of different analytes adsorbed on the Ag NPs films, these were characterized as such by Raman spectroscopy using two excitation lines, 633 and 785 nm. Figure 4 presents the average spectra obtained from the Ag NPs film and from the plastic substrate.

Regardless of the used excitation line, the substrate presented the main Raman bands assigned to polystyrene, which was used as a support for the Ag NPs. These are located at 622, 1001, 1031, and 1602 cm^−1^, among other medium-to-weak intensity bands, and are assigned in the literature to the ring breathing mode of polystyrene, C-H in plane deformation, respectively ring stretching vibrations (Mazilu et al., 2010). The 1001 cm^−1^ band was observed in all spectra acquired from the Ag NPs films, irrespective of the laser line used for excitation. Similar spectra were acquired using the 633 and 785 nm excitation lines, however, better resolved bands and higher quality spectra were obtained under 633 nm excitation. The Raman bands observed in the spectra of the films are mainly assigned to the carboxylate (COO^−^) groups. This was expected, as during the preparation of the Ag NPs, the carboxylate groups of adsorbed citrate ligands remain on the surface of the NPs (Siiman et al., 1983). Moreover, since there are no changes in the SERS band positions or the relative intensities, one may assume that all carboxylate groups of adsorbed citrate ligands are coordinated to surface of the Ag NPs in the deprotonated form (Siiman et al., 1983). The vibrational response in the 1300–1600 cm^−1^ spectral range is consistent with the presence of ionized carboxylate groups from citrate ligands in different geometric and coordination environments. The well-defined band at 1368 cm^−1^ can be assigned to the symmetric deformation vibrations of methylene (−CH_2_) group. The carboxylate coordination to the silver surface can be done in two modes: either monodentate by one oxygen atom through a weak binding or bidentate by both carboxylate oxygen atoms (Siiman et al., 1983). The well-defined band at 1602 cm^−1^ can be assigned to the asymmetric stretching vibrations of (COO^−^) groups. No detectable Raman bands were identified in the spectral region above 1800 cm^−1^ (not shown here). Also note that a good reproducibility of the spectra acquired from the AgNP films was obtained under both excitations, again suggesting a good uniformity of the SERS enhancements across the sample surface. Another concern could relate to the storage stability of SERS substrates, which we verified by analyzingAgNP films after 2 months storage. Their SERS response was very similar to that of freshly prepared AgNP films (Supplementary Figure S2). Although the oxidation of silver surfaces is a real concern, mentioned by several authors in the SERS community, in this case, the ligands on the nanoparticle surface appear to offer some protection against oxidation, and thus prevent aging.

### Assessing the SERS Capabilities of the AgNP Films

First, we assessed the SERS enhancement capabilities of the AgNP films by using a well-known Raman reporter, p-ATP, which binds to the metal surface *via* sulphur atoms, forming a self-assembled molecular monolayer.

**FIGURE 5:**
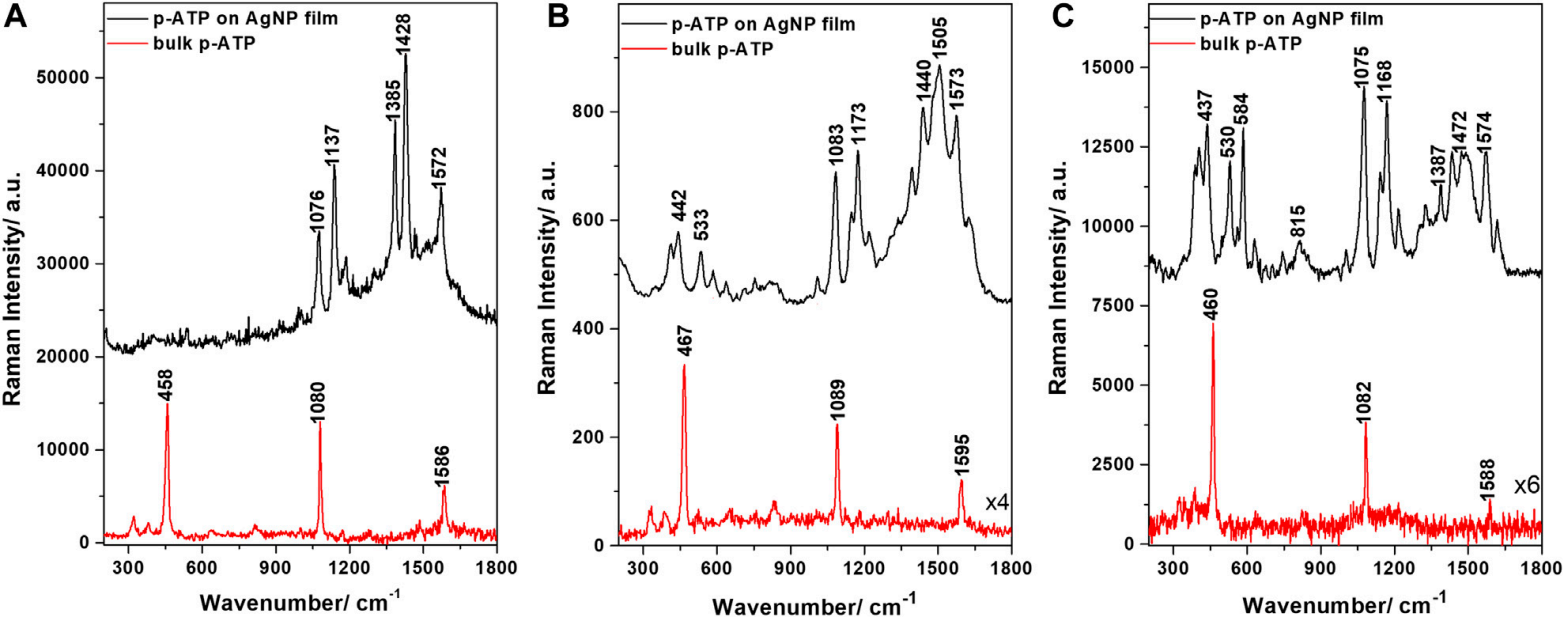
SERS spectra of p-ATP adsorbed on the AgNP film and Raman spectra of bulk p-ATP powder, obtained at different laser excitations: **(A)** 532 nm, **(B)** 633 nm, and **(C)** 785 nm.

Figure 5 shows the typical SERS spectra of p-ATP on the AgNP films recorded at three different commonly employed laser lines, together with the corresponding Raman spectra of p-ATP. A first observation is that the samples are SERS-active at all three laser lines (532, 633, and 785 nm), although showing different efficiencies. The SERS bands observed under 532 nm excitation include those at 1076, 1137, 1385, 1428, and 1572 cm^−1^. A very good quality in terms of signal, signal-to-noise ratio and SERS band density was obtained using the 633 nm excitation line. The p-ATP SERS bands at 533, 584, 1083, 1173, 1241, 1505, and 1573 cm^−1^ are very well defined and clearly enhanced compared with the Raman signal given by the p-ATP bulk powder. For 785 nm excitation, the most intense p-ATP SERS bands are at 530, 1075, 1168, 1326, 1434, 1574, and 1617 cm^−1^. The SERS enhancement factors (EF) were then calculated based on the 1080 cm^−1^ band intensity, the procedure being described in the Supplementary Material. The obtained values were 2.7∙10^3^, 6.6∙10^4^, and 2∙10^5^ for the 532, 633, and 785 nm excitations, respectively. Please note that these are possibly underestimates as opposed to overestimations commonly encountered in the literature. Thus, to conclude this section, the AgNP films exhibited a reasonably good SERS EF at both 633 and 785 nm excitation lines, which will mainly be used for pesticides SERS analyses within the upcoming sections.

### SERS Detection of Thiabendazole Fungicide

**FIGURE 6:**
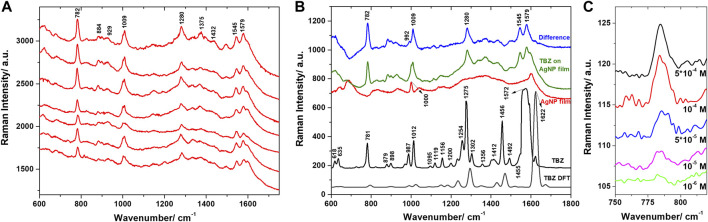
**(A)** SERS spectra of TBZ on AgNPs film collected from various locations on the substrate. Excitation 785 nm. **(B)** The Raman spectrum of TBZ powder and the DFT calculated Raman spectrum compared to the average Raman spectrum of the bare AgNPs film, the average SERS spectrum of TBZ on the film, and their computed difference spectrum. **(C)** SERS spectra of TBZ on AgNPs film for various TBZ concentrations.

The AgNP films were further used for the SERS identification and characterization of TBZ pesticide. Spectra were acquired from various point locations on the samples’ surface and are presented in Figure 6A. The observed spectral features were very similar in all spectra, pointing out the good reproducibility ensured by the developed substrate. The main identified bands are located at 782, 884, 929, 1009, 1280, 1375, 1432, 1545, and 1579 cm^−1^, similar to previous SERS-based identifications of TBZ presented in the literature (Kim et al., 2009; Lin et al., 2018; Oliveira et al., 2020). We calculated the average SERS spectra characteristic to the TBZ on AgNP film, respectively the bare AgNP film and computed their difference spectrum (Figure 6B) in order to identify clearly the TBZ spectral features. Additionally, the difference spectrum is compared to the Raman spectrum acquired from the powder and the DFT calculated TBZ spectrum. For example, following the subtraction, the 1000 cm^−1^ band characteristic to the polystyrene substrate was properly removed and the fingerprint bands of TBZ located around 992 and 1009 cm^−1^ were revealed.

The vibrational spectral analysis was further performed on the basis of the experimental data and DFT calculations. The computed vibrational wavenumbers and Raman activities were employed to identify the vibrational modes explicitly. In consequence, the main bands characteristic to TBZ could be detected in the SERS spectra and assigned to specific vibrations. Good correspondence between the SERS bands and the DFT calculated ones, respectively the Raman bands identified in the spectrum of solid TBZ was revealed.

The bands located at 782 and 1009 cm^−1^ in the SERS spectra show slight enhancement or similar relative intensity to their corresponding Raman bands. The strong 782 cm^−1^ corresponds to the ring breathing mode of benzene ring and C-S stretching vibrations, while the 1009 cm^−1^ shifted from 1012 cm^−1^ in the Raman spectrum is assigned to C-C stretching vibrations and CH bending vibrations in the benzene ring. The 1280, 1545, and 1579 cm^−1^ bands observed in the SERS spectra presented a decreased relative intensity compared to their Raman counterparts. The 1280 cm^−1^ shifted from 1275 cm^−1^ in the Raman spectrum results from C-N stretching of imidazole ring, benzene ring breathing, and weaker contribution from in-plane CH bending vibrations. The 1545 and 1579 cm^−1^ bands are attributed to asymmetric C-C stretching vibrations of the imidazole-thiazole bond, respectively, the benzene ring. Other intense Raman bands, such as the 987 and 1456 cm^−1^, appeared decreased in the SERS spectra. The 1432 cm^−1^ band, shifted from 1456 cm^−1^ in the Raman spectrum, corresponds to C-N stretching vibrations in the thiazole ring, but also C-N, C=C stretching, and CH bending vibrations in the benzimidazole moiety. The 987 cm^−1^ appeared as a shoulder in the computed difference spectrum and is assigned to CH bending and N-C-N bending vibrations.

Most scientific literature in field investigated the TBZ limit of detection in laboratory conditions (Xuan et al., 2019) and tested the possibility of identifying TBZ in various real life samples (Müller et al., 2014; Lin et al., 2018; Fu et al., 2019). Few studies, however, focused on performing a complete vibrational assignment of the TBZ normal modes or on resolving the adsorption mode of TBZ on the metal surface. Oliveira et al. suggested that TBZ interacted with Ag NPs through its thiazole ring based on the assignment of the 782 cm^−1^ band to C-S vibrational modes. Additionally, the local reactivity analysis performed using DFT calculations indicated higher reactivity centred on the sulphur atom (Oliveira et al., 2020). We have seen from our DFT calculations that the 782 cm^−1^ band is assigned mainly to benzene ring stretching vibrations, while the C-S stretching vibrations has a weaker contribution to this normal mode. Given that no other sulphur involving vibrations where observed in the SERS spectra, we can exclude this interaction mode. Kim et al. (2009) investigated the adsorption mechanism of TBZ on a silver mirror in various pH conditions. The enhancement of the out-of-plane vibrations observed at 786, 891, and 1018 cm^−1^ in the SERS spectra, accompanied by the decrease of the ring stretching vibrations 1282, 1458, and 1585 cm^−1^ suggested, according to the SERS selection rules, a parallel orientation of TBZ relative to the silver surface. In acidic pH conditions, however, the enhancement of the 987 and 1631 cm^−1^ bands, which are assigned to C-S, respectively C=N stretching vibrations, indicated that some molecules can adopt a tilted orientation relative to the silver surface and interact *via* the sulphur and nitrogen atoms.

In our case, however, the 782 and 1009 cm^−1^ SERS bands were assigned based on the DFT calculations to in-plane vibrations. According to the SERS selection rules, if a molecule adsorbs through the π electrons to the metal surface, its ring plane would lie parallel to the surface. Therefore, the out-of-plane stretching vibrations would be enhanced, while the totally symmetric in-plane stretching modes would show little to no enhancement. Considering the enhancement of the in-plane vibrational modes observed in our SERS spectra, a rather perpendicular orientation of TBZ molecules to the Ag surface is indicated. Similar observations were presented by Singh et al. (2013) and the authors concluded, based on the binding energies calculated for metal complexes with two TBZ tautomers and the metal-N bonds observed around 230 cm^−1^ that the nitrogen atoms may be involved in the adsorption of TBZ on the metal surface.

**FIGURE 7:**
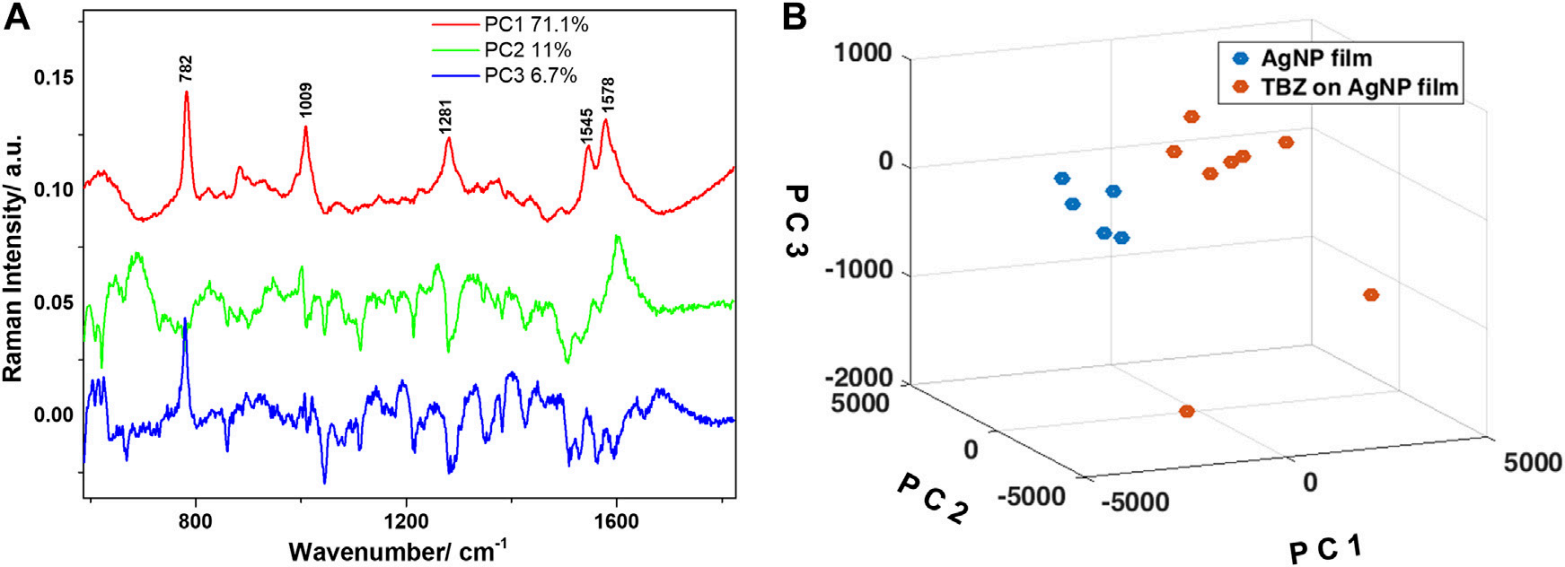
**(A)** Loadings of the first three principal components obtained after PCA applied in the 600–1800 cm^−1^ spectral region. Legend shows the variance captured by each principal component. **(B)** Three-dimensional scatter plot showing the clustering of the spectra collected from the two Ag NPs films as a function of the scores of the first three principal components.

Further, PCA was employed for discriminating between the spectra collected from the bare AgNP film and the film immersed in the TBZ solution. The analysis was applied in the 600–1800 cm^−1^ spectral range, where the main bands of TBZ were detected. The loadings spectra corresponding to the first three principal components (PCs) capturing 88.8% of the spectral variation are shown in Figure 7A. The loadings of PC1 and PC2 captured significant features which could be observed in the computed difference spectrum, as well as the Raman spectrum of the TBZ powder. The main peaks are located at 782, 1009, 1281, 1545, and 1578 cm^−1^ and are identified as SERS spectral components characteristic to TBZ. The loadings of PC3, however, identified a mixture of both TBZ molecular characteristics and the signature of the AgNP film. A proper differentiation of the two groups of spectra was obtained by plotting the scores of the first three PCs (Figure 7B).

Of major concern for practical SERS applications is the possibility to detect pesticides at low concentration. To enquire about the capability of the AgNP films for trace level detection we performed additional measurements on AgNP film exposed to TBZ solutions of different concentrations (5*10^−4^ to 10^–6^ M). We could still observe spectral features of TBZ down to 10^–6^ M concentrations (Figure 6C; Supplementary Figure S3), equivalent to 0.2 ppm, the range relevant for the food industry. In order to verify the sample to sample reproducibility of the AgNP films, three different samples exposed to identical 10^−5^ M TBZ solutions were analyzed (Supplementary Figure S4). Results indicate a good reproducibility of the spectral features observed in the measured SERS spectra. Additionally, the capacity of the AgNP films for thiabendazole SERS-based detection was also demonstrated with a portable Raman spectrometer, results being discussed in the Supplementary Material.

### SERS Detection of the Endosulfan Insecticide

Endosulfan (ES) is an organochlorine pesticide having a poor affinity towards metal surfaces. Therefore, various approaches for capturing ES close to the metal surface have been explored by chemical functionalization of the SERS substrates (Moldovan et al., 2021). Alkane thiol self-assembled monolayer systems offer a versatile method for modifying metal surfaces in order to create molecular pockets between alkyl chains and plasmonic surface (Kubackova et al., 2014). Therefore, ES pesticide molecules could be trapped close to the plasmonic substrate surface through their hydrophobic interaction with alkane thiols, which would allow their detection by SERS (Jiang et al., 2013). The SERS sensitivity is generally influenced by the chain length of alkane thiols (Kennedy et al., 1999). Thus, alkane thiols with different chain length such as hexane thiol (C6, HT) and octane thiol (C8, OT) were used in this work.

**FIGURE 8:**
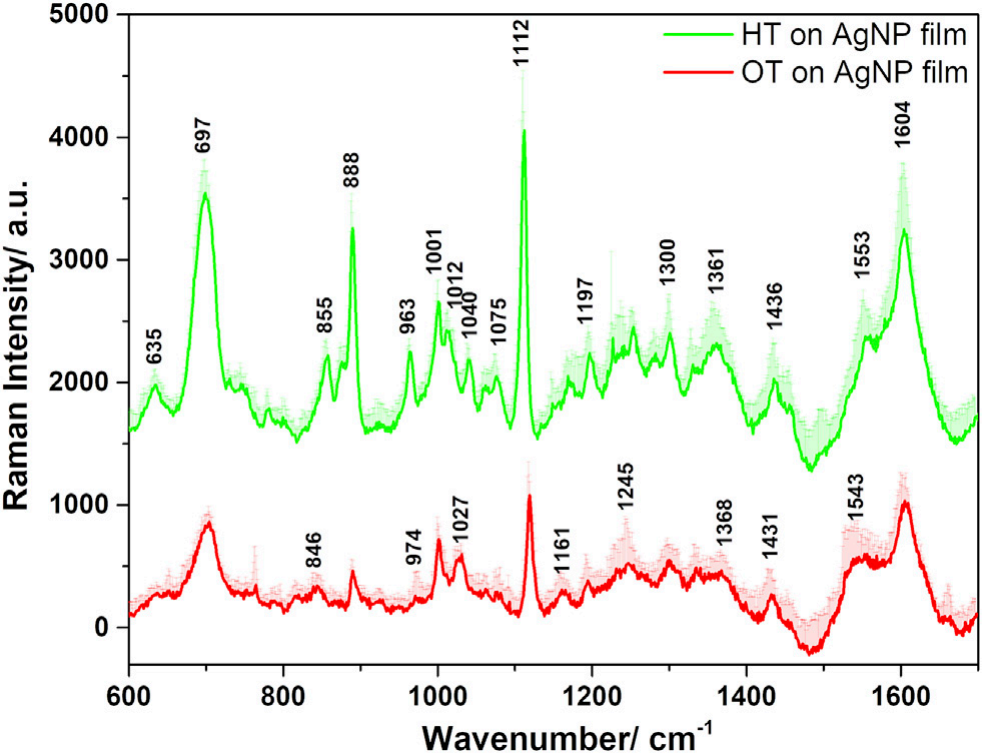
Average SERS spectra of the HT- and OT-functionalized AgNP films plotted together with their positive standard deviations, obtained under 633 nm excitation.

The functionalization of the AgNP substrate with HT and OT was confirmed by the SERS spectra (Figure 8) and their corresponding Raman spectra (Joo et al., 1986; Joo et al., 1987; Kennedy et al., 1999) were used for comparison in this work (spectra not shown here) (John Wiley and Sons, 2022a; John Wiley and Sons, 2022b). Upon adsorption of HT and OT on the AgNP film surface, evidence of their chemisorption is based on the lack of stretching (ν) S-H vibration at about 2575 cm^−1^ (Kubackova et al., 2014). Evidence for the thiolate-Ag bonding is highlighted by the vibrational behaviour in the ν(C-S) spectral region, along with those of the ν(C-C) and ν(C-H) ones. The two ν(C-S) bands for both HT and OT upon adsorption on the Ag surface were observed in the 600–750 cm^−1^ frequency region, being shifted toward much lower frequencies than those in neat alkane thiols (Bain et al., 1989). These vibrations are attributed to “gauche” (G) and “trans” (T) conformers around the two carbon atoms adjacent to the S atom (thiolate group oriented towards the Ag surface). The band at 635 cm^−1^ corresponds to G ν(C-S) and that from 700 cm^−1^ to T ν(C-S) conformers for both HT and OT, the low frequency band being well-defined only in small chain alkane thiolate, such as HT (Joo et al., 1986). However, in the case of AgNP film functionalized with OT these bands were broader and less intense in comparison with those of HT (Joo et al., 1986; Joo et al., 1987; Bensebaa et al., 1999) (Figure 8). The alkane chain adjacent to the C-S bond and presumably near the silver surface adopts mostly the T conformation for both HT and OT.

Stretching C-C vibrations appeared in the 1070–1200 cm^−1^ spectral region and were assigned to either T or G conformation of thiols. Rocking vibrations of methyl and methylene were observed in both HT and OT functionalized AgNP films in the 800–1040 cm^−1^. The wagging and bending methyl vibrations appeared in the wavenumbers spectral region above 1300 cm^−1^ (Supplementary Table S1). The C-H vibrations present a quadratic correlation to the number of carbon atoms in the backbone (Kennedy et al., 1999). The additional two CH_2_ moieties present in OT increase the distance of the methyl group relative to the Ag surface in comparison to the HT case, explaining the decreased signal characteristic to these vibrations. For example, in the case of OT functionalized film, the CH_3_ rocking vibrations presented a lower relative intensity ratio of the 888 cm^−1^ to the 857 cm^−1^ band compared to the HT case, in direct correlation with the influence of the longer alkyl chain length and the hydrophobic interactions between the chains.

The ES pesticide is an asymmetrical molecule containing a rigid norbornene unit connected through a HC_5_-C_6_H bridge to a seven-membered flexible aliphatic ring unit (Supplementary Figure S6), in which only the two methylene (CH_2_) groups and the sulfite (SO_3_) group can induce changes upon its conformation (Schmidt et al., 1997). Therefore, the α- ES conformation is given by the twist chair forms (Schmidt et al., 2001; Schmidt et al., 2014; Schmidt et al., 2017; Guerrini et al., 2008; Hapeman et al., 2013) adopted by the flexible seven-member ring (Schmidt et al., 2017), in which the three protons on the left half are not equivalent with the three protons on the right half of the ring (Schmidt et al., 1997).

**FIGURE 9:**
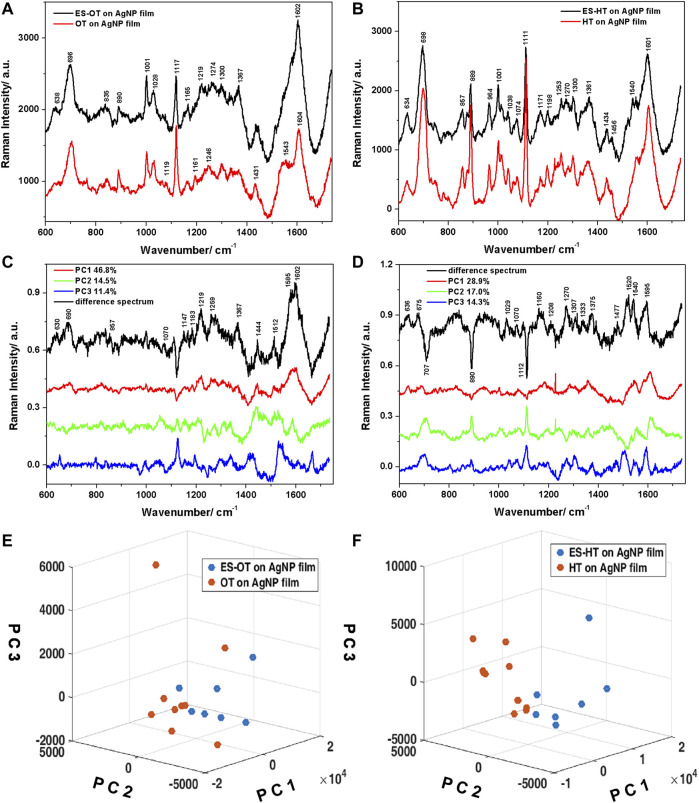
**(A)** Average Raman spectra plotted together with positive standard deviations characteristic to the ES-OT, respectively OT on Ag NPs films. **(B)** Same as **(A)**, for ES-HT, respectively HT on Ag NPs films. **(C,D)** Loadings of the first three PCs and the calculated difference spectra corresponding to the OT, respectively the HT ligands. **(E,F)** Three-dimensional scatter plots showing the differentiation of the two groups of spectra for the OT, respectively the HT case.

The SERS spectrum of ES cannot be seen on Ag NPs, most probably due to the low affinity of the ES towards the rough Ag surface. Thus, the α-isomer of ES was analyzed in its pure form as solid and then as coated onto the AgNP film functionalized with mono-alkanethiols, namely HT and OT. The AgNP films functionalized with endosulfan-octane-thiol (ES-OT) or endosulfan-hexane-thiol (ES-HT) were investigated and the results are presented in Figure 9.

The SERS spectra acquired from the ES-OT (Figure 9A), respectively ES-HT (Figure 9B) functionalized films appeared similar to the ones acquired from the films functionalized only with the respective thiol molecules. At a closer look, however, slight differences can be identified. The SERS spectral data for the coated ES film indicated weakening of some bands associated with Ag bounded monothiols, as well as the appearance of several bands that can be associated with ES. The assignment of the vibrations of ES in both the solid state and onto the AgNP thin films functionalized with HT/OT was performed on the basis of general published data (Guerrini et al., 2008; Schmidt et al., 2014; Schmidt et al., 2017), as well as the theoretical calculations performed in this study. A good agreement between experimental and calculated spectrum of ES was obtained. The calculated ES Raman spectrum data allowed us to complete the assignment of the experimentally observed vibrations. The vibrational peak frequencies and their assignments are given in Supplementary Table S1. Additionally, background interferences from the HT, respectively OT functionalized AgNP films was subtracted from the spectrum of the nanoparticle coated with ES, leading to negative peaks in certain regions of the spectra.

Analysing the SERS spectra acquired from ES-HT AgNP film in comparison to the HT-AgNP film, several band intensities attenuations could be observed. These include, for example, a 10% attenuation of the 700 cm^−1^ band, 40% attenuation of the 890 cm^−1^ band, or 35% attenuation of the 1111 cm^−1^ band. These attenuations were correlated in the difference spectrum with negative peaks around similar values. Additionally, vibrations characteristic to the methyl group of thiols (970–1040 cm^−1^ spectral region) presented attenuated intensity in the SERS spectra of ES functionalized films, which could be explained by the presence of ES molecules hosted within the alkyl chains, thus blocking these associated vibrations. Additionally, the presence of the ES molecules leads to significant attenuation of the C-C skeletal vibrations observed at 1198 cm^−1^ and the CH_2_ wagging vibrations at 1300 cm^−1^, suggesting that the vibrations of thiol alkyl chains are hindered by the ES molecules. Weak ES characteristic bands are identified at 1434, 1456, and 1476 cm^−1^ that can be assigned to CH bending vibrations related to the C_7_-(HC_6_-C_5_H)-C_4_ moiety, the ratio of the 1456 to the 1473 cm^−1^ bands being 3:1, specific to ES molecules (Schmidt et al., 2017). In the OT functionalization case, the presence of ES did not induce significant attenuations of the thiol alkyl chain characteristic bands and ES specific bands are considerably diminished compared to the HT case. Even so, few Raman bands of ES were still observed, such as the weak 1367 cm^−1^ corresponding to twisting vibrations of the C6-C5-C4-H_ax_ moiety.

PCA was applied on the data set consisting of the Raman spectra acquired from the ES-OT and OT functionalized AgNP films. The first three PCs accounted for a variance of 72.8% and the loadings are given in Figure 9C. The PCs were used to differentiate between the two films and the three-dimensional scatter plot showing the separation and clustering of the spectra is presented in Figure 9E. The separation was obtained using mainly PC1, however, PC2 showed a good separation capability, as well. The loading of PC1, which accounted for a variance of 46.8% in the data set, presented similar features to the difference spectrum calculated by subtracting the average Raman spectrum of the OT functionalized AgNP film from the average Raman spectrum characteristic to the ES-OT functionalized AgNP film. The main features included the following Raman bands: 630, 690, 857, 1070, 1147, 1183, 1219, 1259, 1275, 1367, 1444, 1512, 1585, and 1602 cm^−1^. The behaviour of the seven-membered flexible ring of ES can be described from our SERS ES-OT spectra by the skeletal deformation vibrations observed at 690 cm^−1^, CH deformation vibrations at 857 and 1070 cm^−1^, the H-C5-C6-H wagging vibrations at 1147 cm^−1^, and the CH_2_ bending vibrations at 1444 cm^−1^. Moreover, we identified the out-of-plane twisting vibrations of the C6-C5-C4-H_ax_ moiety at 1367 cm^−1^. Additionally, symmetric, respectively asymmetric stretching vibrations of the dialkylsulfite group (C-SO_3_-C) were found at 1183 and 1219 cm^−1^. The broad band observed at 1580–1600 cm^−1^ in both the calculated difference spectrum and the loadings of PC1 may be assigned to combined stretching vibrations of C5-C and C6-C, respectively deformations of C6H and C5H of ES (Schmidt et al., 2017).

Similar analysis was applied to the ES-HT functionalized AgNP film (Figures 9D,F). The first three PCs used for clustering the two groups of spectra accounted for 60% of the total variance in the data set. Although PC1 explained almost 30% of the total variance, PC2 presented negative contributing peaks, which were identified in the calculated difference spectrum (average spectrum characteristic to the HT on AgNP film subtracted from the average spectrum corresponding to the ES-HT on AgNP film) and allowed the best differentiation between the two groups of spectra. The main bands observed in the computed difference spectrum are located at 636, 675, 1029, 1070, 1160, 1208, 1270, 1307, 1372, 1476, 1520, 1540, and 1595 cm^−1^. The SERS behaviour of ES on the HT-AgNP film can be explained mainly by the skeletal deformation vibrations observed at 636 and 675 cm^−1^, CH wagging and deformation vibrations located at 1029 and 1476 cm^−1^, as well as in-plane and out-of-plane twisting vibrations of the moiety containing H-C5-C6-H bridge in the 1330–1380 cm^−1^. Also, combined stretching and deformation vibrations of the H-C5-C6-H bridge connecting with the norbornene unit appeared at 1595 cm^−1^.

Our results indicate that the longer alkane chains of OT allowed a more efficient trapping of the ES molecules compared to the shorter HT chains, leading to a better SERS response. Due to the weak interaction between the alkane chains from thiol layers, their assembly is rather compact, reducing the possibility of a direct interaction of the pesticide with the metal surface. However, the adsorbed HT should present a more disordered structure compared to OT, which has a longer chain and favours an increase of the aliphatic chain order (Kubackova et al., 2014). The more organized OT layers could lead to a better retention of the ES molecules near the SERS substrate.

Several analytical methods can be used for detection and identification of pesticides such as thiabendazole and endosulfan: gas chromatography (GC) (Hong et al., 2004; Liu et al., 2016), liquid chromatography-mass spectrometry (LC-MS) (Famiglini et al., 2008; Ferreira et al., 2016), high-performance liquid chromatography (HPLC) (Uclés et al., 2015), and HPLC combined with fluorescence detection (Liu et al., 2012), and ultraviolet detection (Wang et al., 2018). Nonetheless, these techniques imply laborious sample preparation, time-consuming detection procedures, moreover, they require operational skills and expensive equipment or agents that limit their further applications for rapid and *in situ* applications for TBZ and α-endosulfan detection. To overcome these limitations, SERS has been successfully implemented in environment applications. Its fingerprinting ability enables the specific and rapid identification of pesticides in a non-destructive, non-invasive manner with no or simple sample preparation/manipulation as well as low sample volume (Alsammarraie et al., 2018; Li et al., 2021). Compared to other analytical methods, SERS is an ultrasensitive tool demonstrating its excellent sensitivity not only for the detection of TBZ and α-endosulfan but for a wide range of pesticides (Liu et al., 2013; Pang et al., 2016). Therefore, the implementation of SERS in environmental applications is greatly desired and could have a great impact on pesticides monitoring activities.

## Conclusion

SERS substrates were fabricated by convective self-assembly of colloidal silver nanoparticles on solid planar substrates. Dense, highly compact silver nanoparticle films exhibiting a high density of SERS hot-spots, were obtained for the first time on mm^2^ areas. This approach, relying on evaporation-induced colloidal self-assembly does not imply any complicated surface chemistry protocols, and the methodology can be extended to a broad range of chemically synthesized colloidal nanoparticles in aqueous media. The fabricated AgNP film SERS substrates were tested with various kinds of molecules, such as para-aminothiophenol, thiabendazole, α-endosulfan, and alkanethiols, demonstrating the SERS efficiency of these SERS substrates for a broad range of analytes. The SERS enhancement factors of the fabricated AgNP films were evaluated using the well-known Raman probe p-aminothiphenol, exhibiting the largest SERS enhancement factors for 785 nm, reaching values larger than 10^5^. The capability of these AgNP films for the SERS analyses of two kinds of pesticides, the fungicide thiabendazole and the insecticide α-endosulfan were then demonstrated. Thiabendazole could be readily adsorbed on the AgNPs without any sample surface functionalization and detected down to 10^−6^ M, which falls in the sub-ppm range. The reached sub-ppm range is comparable to the range reached by other SERS studies, however with a SERS substrate that is relatively simple to fabricate. Since TBZ contains S and N atoms which can act as specific binding sites for noble metal SERS surfaces, the obtained results may pave the way towards the detection of other pesticides with similar chemical structure. Endosulfan is a very challenging analyte, due to its poor affinity to metal surfaces, which is also the reason why scarce results can be found in the literature. We managed to capture it near the metal surface by using self-assembled alkane thiol monolayers (hexanethiol and octanethiol), as demonstrated by the thorough vibrational band analyses, and supported by DFT calculations. Several vibrational bands that can be attributed to endosulfan were identified by analyzing the spectral differences between the thiol-functionalized SERS substrate before and after exposure to endosulfan. The potential of Principal Component Analysis of SERS spectra for pesticides identification was also highlighted. Overall, these results constitute a step towards the development of SERS-based sensors for pesticides detection, identification and monitoring.

## Data Availability

The original contributions presented in the study are included in the article/Supplementary Material, further inquiries can be directed to the corresponding author.
