# Supplementary material for: Silver Nanoparticle Films Obtained by Convective Self-Assembly for Surface-Enhanced Raman Spectroscopy Analyses of the Pesticides Thiabendazole and Endosulfan
Source: Front Chem. 2022 Jun 29;10:915337. doi: 10.3389/fchem.2022.915337 (PMC9277229; doi:10.3389/fchem.2022.915337)
Supplement: Supplementary file 1 [file DataSheet1.PDF]

Supplementary Material

1 Ag nanoparticles size distribution

The size distribution of the Ag nanoparticles used in this study was estimated based on TEM images. The histogram in Supp. Fig. 1 indicates a bimodal size distribution, the majority of particles having sizes around 40 nm, with an additional population sized around 60 nm.

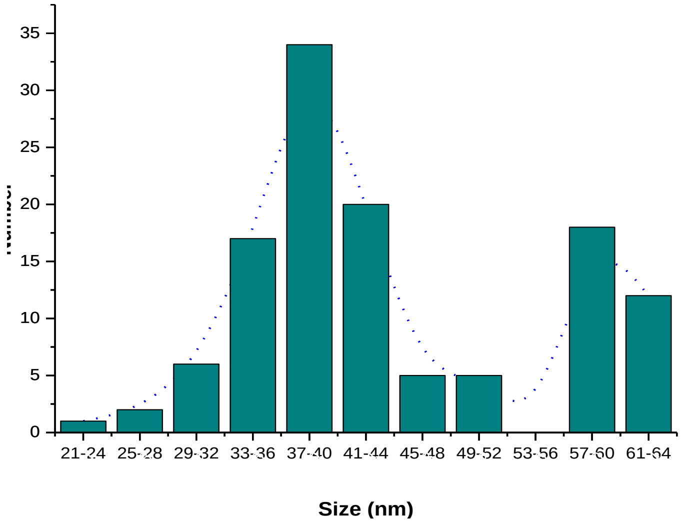

Supplementary Figure 1. Size distribution of the synthesized colloidal Ag nanoparticles. The blue line fit serves as a guide.

2 Storage stability of AgNP films

The storage stability of the AgNP films was evaluated by measuring the Raman/SERS response of a freshly fabricated empty/bare AgNP film with that of a two-months old sample. Results are displayed in Supp. Fig. 2.

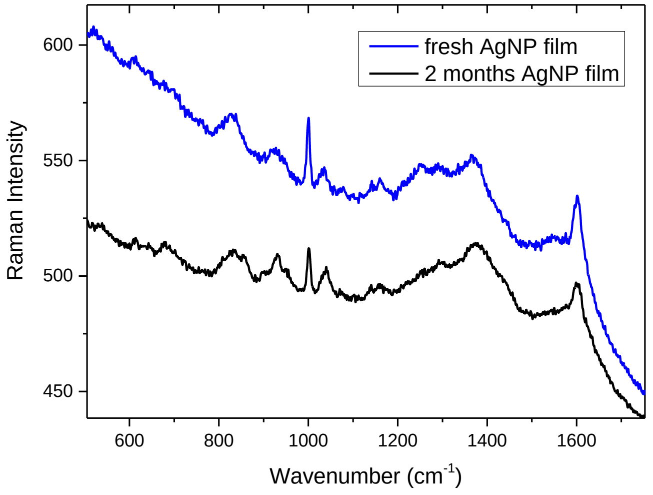

Supplementary Figure 2. Raman/SERS spectra of a fresh and two-months old bare AgNP film.

3 SERS EF calculation

The SERS enhancement factor (EF) was calculated according to the formula:

$$EF = \frac{I_{\mathrm{SERS}}/N_{\mathrm{SERS}}}{I_{\mathrm{Raman}}/N_{\mathrm{Raman}}}$$

whereI_{SERS} is the intensity (integrated band area) of the SERS band at 1080 cm^{-1}, N_{SERS} the number of excited molecules on the SERS substrate surface, I_{Raman} the intensity of the same band in the Raman spectrum, and N_{Raman} is the number of molecules in the volume of the focus. Other values involved in the EF calculation are given in Table S1, for all three wavelengths of excitation (λ_{exc}): D_f - diameter of the focus spot, A_f - area of the focus spot, H_f - height of the focus (two times the Rayleigh range), V_f - volume of the focus, approximated by a cylinder of diameter D_f and height H_f, M_f - the mass of pATP in the focus. For 532 nm a 100× objective was used, while for 633 and 785 a 20× one.

Table S1. Different values involved in the calculation of the SERS EF.

λ_{exc} (nm)	D_f (μm)	A_f (μm)	H_f (μm)	V_f (μm^3)	M_f (g)	N_{Raman}	N_{SERS}	I_{Raman}	I_{SERS}	EF
532	0.76	0.45	1.72	0.79	9.30E-13	4.47E+9	2.28E+6	1.82E+5	2.50E+5	2.69E+3
633	1.93	2.92	9.24	27.05	3.20E-11	1.54E+11	1.46E+7	1.21E+3	7.71E+3	6.65E+4
785	2.39	4.49	11.46	51.59	6.08E-11	2.93E+11	2.25E+7	1.13E+4	1.75E+5	2.03E+5

4 SERS spectra of AgNP films exposed to different TBZ concentrations

The whole spectral range of the SERS spectra of TBZ at different concentrations is presented in Supp. Fig. 3.

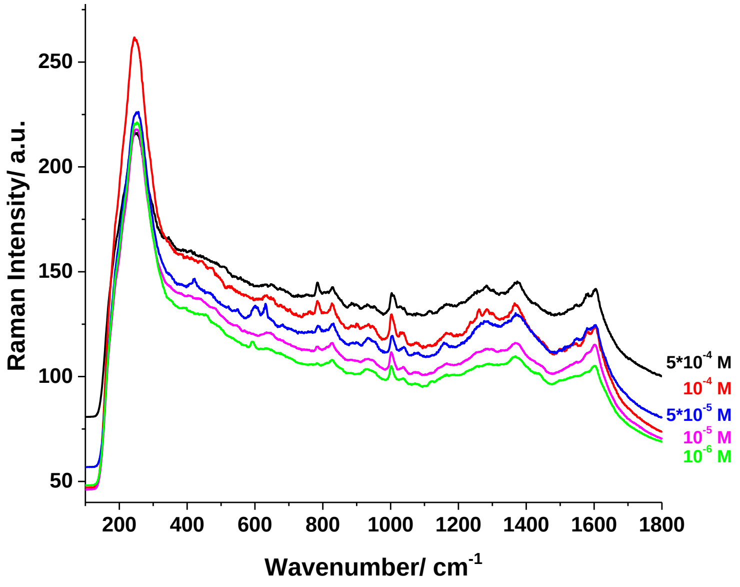

Supplementary Figure 3. SERS spectra ofAgNP film exposed to TBZ solutions of different concentrations ($5*10^{-4}$ to 10^{-6} M).

5 Sample to sample reproducibility

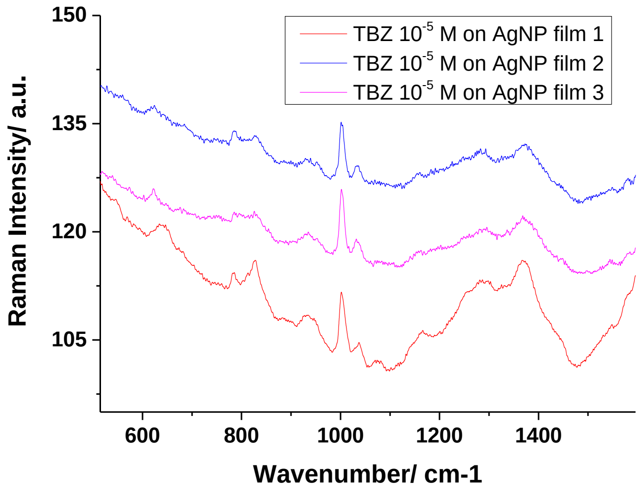

Supplementary Figure 4. SERS spectra of TBZ measured on three different AgNP films.

6 SERS of thiabendazole with a portable Raman spectrometer

The aim of this experiment was to determine if the Ag NPs films could be employed for future on-site experiments. In such applications compact and portable Raman systems need to be used, which are not as performant as benchtop Raman spectrometers. Several spectra acquired from the TBZ adsorbed on the Ag NPs film are presented in Supp. Fig. 5. The laser line employed for excitation was 532 nm and even if the Ag NPs films showed the lowest enhancement factor for this excitation wavelength, TBZ could be detected. The fingerprint band of TBZ at 776 cm^{-1} was observed in all spectra collected from various locations of the AgNP film. Moreover, a good reproducibility of the SERS spectra can be observed. Other TBZ characteristic bands identified in the spectra are located at 1271, 1449, and 1572 cm^{-1}. Additionally, two weak bands are detected at 985 and 1003 cm^{-1}, assigned to the thiazole ring breathing, respectively, the C-C stretching of the benzene ring. Their unambiguous identification, however, is hindered due to overlapping caused by the 995 and 1024 cm^{-1} bands observed also on the bare film. As we have seen from the 785 nm measurements presented in the main text, these bands are characteristic to the polystyrene supporting substrate. Therefore, a better identification of the two TBZ bands located in this spectral region is obtained following the subtraction of the Ag NPs film from the spectrum of the film immersed in the TBZ solution (not shown here). The good reproducibility of the SERS spectra characteristic to the TBZ adsorbed on the Ag NPs films obtained in this study suggests that these films can be used for its detection even under 532 nm laser line excitation, despite the lower amplification presented by the substrates under these conditions. Moreover, it suggests that the AgNP films could be used to detect TBZ pesticide on-field, at possible contaminated locations.

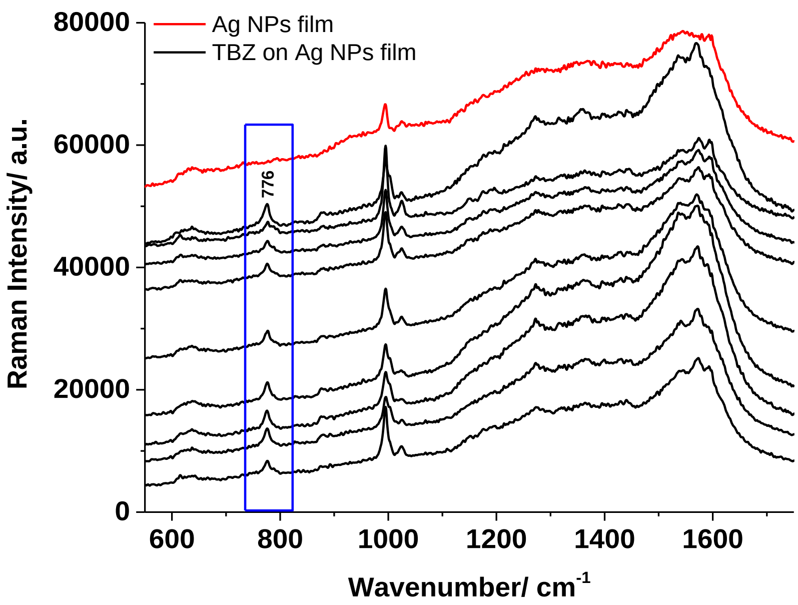

Supplementary Figure 5. SERS spectra of TBZ on the AgNP film, and of the bare AgNP film, recorded using a portable Raman spectrometer under 532 nm excitation.

7 Molecular structure of α-endosulfan

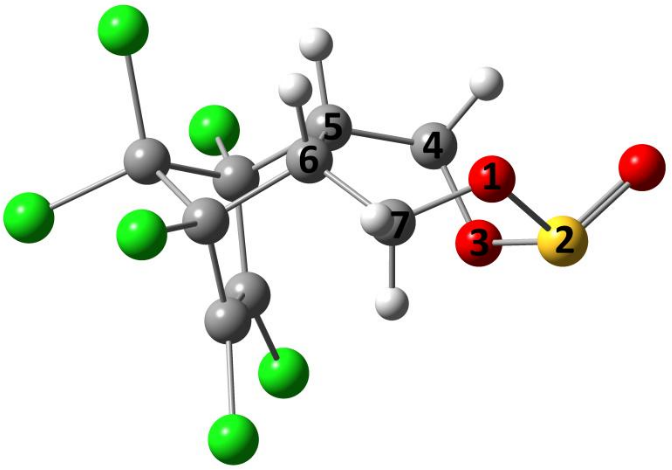

Supplementary Figure 6. The equilibrium geometry of α-endosulfan, obtained by DFT computation, including numbering of some atoms as used in the main text. Red- oxygen atoms, yellow- sulphur atom, green- chloride atoms, dark gray – carbon atoms, white- hydrogen atoms.

The seven-membered ring in the α-endosulfan molecule numbering used here is: the ring oxygen atoms are atoms 1 and 3, sulphur is atom 2, the methylene carbons are C4 and C7, and the methine carbons are C5 and C6 (see Supp. Fig. 6).

8 Band assignment for endosulfan on OT-and HT-functionalized AgNP films

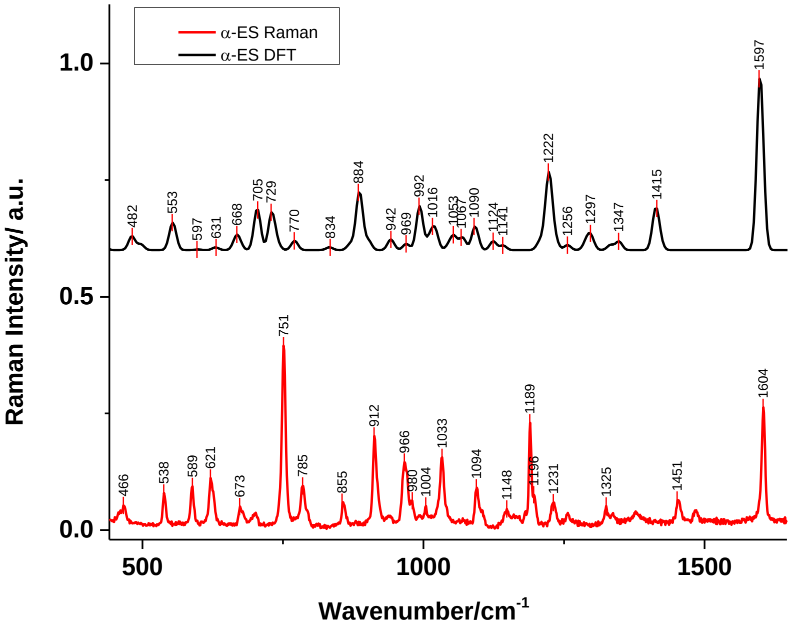

Supplementary Figure 7. Experimental and DFT calculated Raman spectra of α-endosulfan

The experimental and theoretically obtained Raman spectra of α-endosulfan are given in Supp. Fig. 7. Table S2 presents the complete vibrational assignment of α-endosulfan, HT and OT adsorbed on the AgNP films, and of ES-HT, respectively ES-OT adsorbed on the AgNP films. Highlighted in blue are the bands assigned to endosulfan with most confidence.

Table S2. SERS band positions and vibrational assignment of HT and OT adsorbed on the AgNP films and of the ES-HT, respectively ES-OT adsorbed on the AgNP films.

**Wavenumber (cm^{-1})**						**Vibrational assignment of HT and OT**
**α-ES**		**HT on Ag NPs film**	**αES-HT on Ag NPs film**	**αES-OT on Ag NPs film**	**OT on Ag NPs film**	
**Experimental**	**Theoretical**<br>**Vibrational assignment**					
621 m	629 vw<br>ω8 asym	635 m	634 w	638 w	633 w	ν (C-S) G
673 w	668 w<br>ω7 sym re			668 w		
701 vw		697 s	698 s	696 m	704 m	ν (C-S) T
751 vs	705 m<br>ρ(C4H_2)/<br>ν (=C-C_l) (Sreekumaran Nair et al., 2003)					
785 m	729 m<br>ρ(C7H_2)re					
855 w	770 w<br>ρ(C7H_2) rc	855 w-m	857 w-m	835 vw	846 vw	
		875 vw	874 vw	874 w	877 vvw	
		888 m-s	889 m	890 w-m	888 m	ρCH_3 T
912 m	884 m<br>ν(C-Cl) + ω(CH_2)		916 w			

966 m	992 m<br>Ring breathing	963 m	964 w-m	975 vw	974 vw	ρCH_3 T
1004 vw		1001 m-s	1001 m	1001 m	1001 m	ρCH_3 T
1033 m	1016 w<br>ν(C-C) + $\omega(CH_2)$	1040 w	1038 w	1028 m	1027 m	ρCH_3 T
1094 m	1090 w<br>(C-C) + δ(CH)	1075 w	1074 m	1070 vw	1075 vw	ν (C-C) G
-		1112 vs	1111 s	1117 m	1118 m	ν (C-C) T
1148 vw	1141 w<br>ω_{HH} (H_{eq}-C7-C6-H_{eq})<br>ω_{HH}(H_{ax}-C5-C4-H_{ax})			1147 vw		
1189 s	1222 s<br>ν(S=O) + ω_{HH}(H_{ax}-C5-C4-H_{eq}) +ω_{HH}(H_{eq}-C7-C6-H_{ax})	1168 w	1171 m	1165 w	1161 w	ν(SO) in SERS
1196 vw (sh)		1197 w	1198 w	1196 vw		ν (C-C) T+
1231 w	1256 w<br>ω_{HH}(H_{ax}-C7-C6-H_{eq})			1219 w		ν (C-O-SO-O-C)
1256 vw		1253 w-m	1253 w-m	1247 w	1245 w	
			1270 w-m	1263 w		

-		1300 w-m	1300 w-m	1300 w	1300 w	ω (CH_2)
1325 vw	1297 vw<br>γ ip (H_{eq}C7-C6-C5)<br>γ ip (C6-C5-C4-H_{eq})	1330 vw	1333 vw	1333 vw	1335 vw	ω (CH_2) in SERS
-		1361 w-m	1367 w-m	1367 w-m	1360 w	δ(CH_3) sym
1380 vw	1347 w		1376 w (sh)	1378 w (sh)		γ op (C6-C5-C4-H_{ax})
		1436 w-m	1434 w-m	1434 w-m	1431 w-m	
1451 w	1415 m<br>δ C-H ip(C4H_2)H_{ax}, H_{eq}, (C7H_2)H_{eq}	1459 w	1456 w<br>δ(CH_3) asym +<br>δC-H ip(C4H_2 H_{ax}, H_{eq}, C7H_2 H_{eq})	1444 vw<br>δ(CH_3) asym +<br>δC-H ip(C4H_2 H_{ax}, H_{eq}, C7H_2 H_{eq})	1461vw	δ(CH_3) asym
1484 vw	1421 w<br>δ C-H op (C7H_2)H_{ax}		1476 vw			
-			1521 vw	1514 vw		
		1553 w	1540 w<br>1553 w	1541 vw<br>1577 w sh	1543 w	
1604 s	1597 s<br>ν(C-C6) + ν(C6-C5)+ ν(C5-C)+ρ(C6H)$_{ip}$+ ρ(C5H)$_{ip}$	1604 s	1601 s	1602 s	1604 s	ν (COO^-) asym. from citrate

vw- very weak, w-weak, m-medium, s-strong, ν-stretching, δ-bending, γ-twisting, ω-wagging, ρ-rocking, sym-symmetric, asym-asymmetric, T-*trans* conformer, G-*gauche* conformer, ip-in-plane, op-out-of-plane, re-ring expansion, rc-ring compression

References

A. Sreekumaran Nair, T. Renjis Tom, T. Pradeep, Detection and extraction of endosulfan by metal nanoparticles, *J. Environ. Monit.*, 2003, 5, 363-365.
